# Availability optimization of power generating units used in sewage treatment plants using metaheuristic techniques

**DOI:** 10.1371/journal.pone.0284848

**Published:** 2023-05-04

**Authors:** Monika Saini, Ashish Kumar, Dinesh Kumar Saini, Punit Gupta

**Affiliations:** 1 Department of Mathematics & Statistics Manipal University Jaipur, Jaipur, India; 2 Department of Computer & Communication Engineering, Manipal University Jaipur, Jaipur, India; 3 School of Computer Science, University College Dublin, Dublin, Ireland; Univerzitet Singidunum, SERBIA

## Abstract

Metaheuristic techniques have been utilized extensively to predict industrial systems’ optimum availability. This prediction phenomenon is known as the NP-hard problem. Though, most of the existing methods fail to attain the optimal solution due to several limitations like slow rate of convergence, weak computational speed, stuck in local optima, etc. Consequently, in the present study, an effort has been made to develop a novel mathematical model for power generating units assembled in sewage treatment plants. Markov birth-death process is adopted for model development and generation of Chapman-Kolmogorov differential-difference equations. The global solution is discovered using metaheuristic techniques, namely genetic algorithm and particle swarm optimization. All time-dependent random variables associated with failure rates are considered exponentially distributed, while repair rates follow the arbitrary distribution. The repair and switch devices are perfect and random variables are independent. The numerical results of system availability have been derived for different values of crossover, mutation, several generations, damping ratio, and population size to attain optimum value. The results were also shared with plant personnel. Statistical investigation of availability results justifies that particle swarm optimization outdoes genetic algorithm in predicting the availability of power-generating systems. In present study a Markov model is proposed and optimized for performance evaluation of sewage treatment plant. The developed model is one that can be useful for sewage treatment plant designers in establishing new plants and purposing maintenance policies. The same procedure of performance optimization can be adopted in other process industries too.

## 1. Introduction

Water is the precious commodity on earth for the survival of living creatures. It is as important as air, and no one can imagine life on earth without water. On the earth, 71% surface is covered by water, among which 96.5% is stored in oceans. Till now, instead of technological advancements, human being is not in a position to use ocean water for drinking and agriculture purposes. Only a limited amount of water is available which can be used for drinking and agriculture. Water resources are very limited, and many areas in various countries depend on rain and rivers’ water supply. Countries like India, which accommodate 16% of the world population while only 4% of water resources, face challenges in providing drinking water for their citizens as ground water level is very low in some areas below 600 feet. In this challenging scenario, it becomes important to use water resources with great care, and simultaneously such techniques will be developed through which used wastewater can be recycled.

Many researchers are consistently working in this direction, and significant growth has been observed in the field of wastewater treatment by establishing sewage treatment plants. Sewage treatment is the procedure of removing pollutants from wastewater produced by households and industries. Sewage treatment is a three-stage procedure having physical, chemical, and biological stages. The water treated through sewage treatment is safe for the environment and can be utilized for agriculture, while semi-solid sludge can be decomposed either in land or used for energy generation in the form of methane gas. Sewage treatment plant has a very complex design, and it is assembled using many subsystems/components. The sewage treatment plant’s complexity influences the system’s operational performance, and it becomes necessary to operate these plants with utmost care. This can be achieved by the reliability and availability of the plants. As the sewage treatment plants for other industries like process industries, mechanical systems, production lines, transport industries and network systems, reliability and availability are key performance measures for successful operation.

Keeping in mind all the above facts, the present study is designed to research the provision of strength-producing units in sewage remedy plants. In the existing study, an attempt has been made to provide a unique mathematical version for strength-producing unit assembled in sewage remedy plants. Markov birth-death of life technique is followed for version improvement and technology of Chapman-Kolmogorov differential-distinction equations. The worldwide answer is found in the use of metaheuristic strategies particularly genetic algorithm (GA) and particle swarm optimization (PSO). All time-established random variables related to failure charges are taken into consideration and exponentially dispensed even as restore charges comply with the arbitrary distribution. The restore and transfer gadgets are best and random variables are unbiased to every other. The numerical consequences of gadget availability had been derived for exclusive values of crossover, mutation, several generations, damping ratio, and population size to attain optimum value. The results were also shared with plant personnel. Statistical investigation of availability results justifies that PSO outdoes GA in predicting the availability of power-generating systems. The findings of the present study will be very useful for sewage treatment plant designers in establishing new plants and purposing maintenance policies. The proposed methodology and algorithms can be utilized in other production and process industries like Paper and Pulp, Shoe Manufacturing, Sugar Industry, Sewage Treatment Plant, etc., to optimize the performance of the systems. In short, the main contributions of present study are as follows:

- Development of mathematical model for power generating unit of sewage treatment plant
- Etimation the best values of failure and repair rates using GA and PSO
- Optimization of mathematical model using GA and PSO and prediction of optimal availability

This complete study is divided into eight sections, including an introduction and detailed literature review presented in section 2. Section 3 discusses the system description. Section 4 material and methods in which some relevant definitions are appended. The proposed mathematical model is presented in Section 5. Experimental results and optimization strategies and their implementation are appended in Sections 6 and 7, respectively. Concluding remarks and future directions are discussed in Section 8.

## 2. Literature review

Several studies have been conducted on design perspectives and the establishment of sewage treatment plants. Olsson (1976) [1] presented the state-of-the-art design of sewage treatment plants to control their failures and enhance operationality. Berthouex et al. [2] (1978) investigated some quality aspects of monitoring the sewage treatment plants. Boger [3] (1992) explored the applicability of neural networks in the operation of wastewater treatment plants. Wang and Pham [4] (1999) proposed various maintenance models for production industries using the concept of imperfect maintenance. Li and Pham [5] (2005) discussed the effect of random shock and multi-component failure on degraded systems reliability. Amari et al. [6] (2006) used the Markov process to develop an industry’s cost-effective maintenance schedule. Pham [7] (2006) described the important concepts used in reliability modelling.

Ling and Isa [8] (2006) suggested bioremediation of oil sludge contaminated soil by using sewage sludge in the fields. Mjalli et al. [9] (2007) used an artificial neural network and black-box modelling to predict wastewater treatment plants’ performance. Yang et al. [10] (2010) developed the assessment system for measuring operational energy performance in wastewater treatment plants. Manzini et al. [11] (2010) suggested various maintenance policies for industrial systems. Wang and Pham [12] (2011) modelled dependent competing risks having multiple degradations and random shock using copulas. Amari et al. [13] (2012) used warm standby redundancy in k-out-of-n systems. Malhotra and Negi [14] (2013) used particle swarm optimization (PSO) in reliability investigation. You and Pham [15] (2016) conducted the reliability evaluation of a CNC system using the field data. Mannina et al. [16] (2016) presented a detailed review of the tools for measuring greenhouse gases from wastewater treatment plants.

Pham [17] (2016) suggested applications of computing in reliability management. Duan et al. [18] (2017) discussed a model for recovering thermal energy from small-scale sewage treatment plants situated in northern Canada. Gautam et al. [19] (2017) developed a cost-effective treatment technology for small sewage treatment plants in different parts of India. Zhu and Pham [20] (2018) used the martingale process with Gamma distributed environmental factors in software reliability evaluation. Xie et al. [21] (2018) proposed an efficient stochastic model for hybrid-electric buses predicting energy management with reference to the state-of-charge advisory. Olyaei et al. [22] (2018) developed a system for assessing flood reliability due to wastewater treatment plants. Mlynski et al. [23] (2019) investigated the applications of mathematical simulation methods for assessing the operational reliability of wastewater treatment plants. Boyd et al. [24] (2019) discussed the flowing in forecasting for wastewater treatment plants. Pham and Pham [25] (2019) proposed a general reliability model using a stochastic fault-detection rate. Lin et al. [26] (2019) performed a reliability evaluation of a multi-state air transport network system using the concept of multiple demands. Gu et al. [27] (2019) used copula methodology for reliability calculations of mechanical systems under dependent failure mechanism. Chang [28] (2019) used a simulation approach for the reliability estimation of a stochastic production system. Lin and Chen [29] (2020) used the flow data mining technique in the reliability evaluation of multistate networks. Huang et al. [30] (2020) discussed the impact of multiple terminals under stocks for the reliability investigation of multi-state distribution networks. Zhu and Pham [31] (2020) used stochastic modelling in the development of software reliability model. Lee et al. [32] (2020) discussed the concept of dependent failure and SPRT in software reliability. Mesquita et al. [33] (2021) developed reliable technologies for assessing the feasibility of biogas use in sewage treatment plants. Al Abdali et al. [34] (2021) carried out a reliability analysis of blowers used in sewage treatment plants. Niu et al. [35] (2021) studied a multi-state system’s reliability under the concept of cost and spoilage. Zhu [36] (2021) proposed a new model of complex systems’ reliability evaluation under an imperfect maintenance strategy. Ostadi and Hamedankhah [37] (2021) suggested a two-phase reliability optimization methodology for series-parallel systems. Piri et al. [38] (2021) analyzed pumping stations’ reliability for sewage networks using a hybrid neural network and genetic algorithm. The applicability of metahurisitic approaches observed in various files like environment [39–42], energy [43,44], and business [45–47].

Sinwar et al. [48] (2021) used GA and PSO for the availability and performance investigation of physical processing units in the sewage treatment plant. Kumar et al. [49] (2022) used metaheuristic approaches in the optimization of operational availability of cooling towers of STTPs. Saini et al. [50,51] optimized the availability of condenser of STTPs, and biological and chemical units of treatment plants using evolutionary and swarm-based algorithms. It is observed that many researchers carried out studies related to the design of sewage treatment plant, but the reliability aspects of plants as well as the power generating unit is not so extensively discussed so far in the literature.

## 3. System description

Two factors, domestic uses and industrialization, are mainly responsible for polluting water bodies. Solid waste and chemicals from industries are drained from industries into water bodies. The wastewater generated through domestic use can also be recycled. The power generating unit is studied out of the four main units, physical processing unit, biological and chemical processing unit, power generation processing unit, and sludge digestion processing units. The power generation processing unit is used to generate energy by treating the remaining sludge in the finalized stage of this treatment. For this, it incorporates in six subsystems as sludge digesting units, which lessen the quantity of sludge and shape biogas like methane and carbon dioxide, fueloline maintaining tank saved gases and stability the fluctuation withinside the manufacturing of biogases in digester and burner disposed extra and undesirable gases from the system. Gas scrubber eliminates hydrogen sulfide, neutralize dangerous components, and soak up pollutant from this, and fueloline engine runs on gaseous gasoline and a heated digester. In the last level electricity is generated with the assist of fueloline engine, and all STP units function. Sludge digesters, in addition to the electricity era, include a unit configured as 2-out-of -2: G, at the same time as fueloline maintaining tank, fueloline burner, fueloline scrubber and fueloline engine are composed of unmarried units.

## 4. Statistical analysis

### 4.1 Mann-Whitney U-test

Mann-Whitney U-test is used to test the equality of two population means when sample size is small and normality is not attained. Suppose *x*_1_, *x*_2_, ……‥, *x*_*m*_ a sample taken from a population having cumulative density function (c.d.f) *F*_*x*_(*x*) and another sample *z*_1_, *z*_2_, ……‥, *z*_*n*_ has been taken from another population with c.d.f. *F*_*z*_(*z*). The populations do not follow the normal distributions. If we want to test the significance *H*_0_:*F*_*x*_(*x*) = *F*_*z*_(*z*) against an alternative hypothesis, *H*_1_:*F*_*x*_(*x*) ≤ *F*_*z*_(*z*) then Mann-Whitney U-test is the most appropriate test for it. Here, U-statistic is a measure of the difference between the ranked observations of the two samples. For comparison, the global output of metaheuristic approaches to non-parametric tests is always recommended as parametric test assumptions are not satisfied.

### 4.2. Stochastic differential difference equations using markov process

A stochastic process is called the Markov process if its dynamic behaviour is such that the probability distribution for reaching the next state only depends on the present state, not on how it arrives at the present state. In formulating a Markov model, it is necessary to define all the states of the model. If the hazard rate between two states is constant, the model is homogenous; otherwise, it is nonhomogeneous.

### 4.3Assumptions

1. Sufficient repair facility is available in the plant immediately.
2. Distribution for the failure rate and repair rates are considered exponentially distributed.
3. System performs as new with full capacity after the repair.
4. Switch devices are perfect.
5. No occurrence of simultaneous failures.

### 4.4 Simulating environment

For simulating the experiments, we used MATLAB R2019a on the Windows 10 64-bit operating system with 8 GB of RAM and an Intel Core i5 8th generation CPU. Initially, random samples are generated using exponential distribution and then GA and PSO algorithms applied to obtain the optimal solution.

### 4.5 State transition diagram

**Fig 1:**
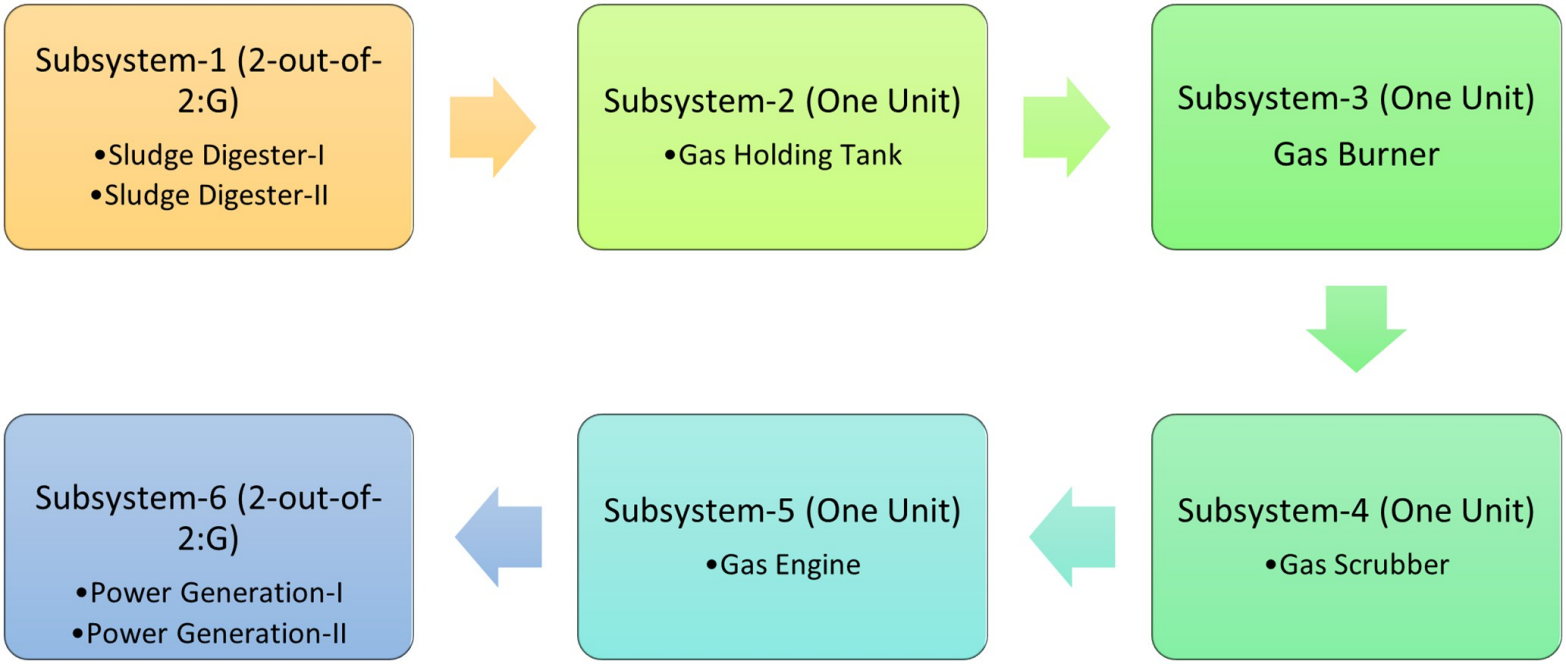
Configuration diagram of power generating unit.

In this section, a state transition diagram of power generating unit is designed by considering exponentially distributed failure and repair rates based on the configuration of system as given in Fig 1.

### 4.6 Failure and repair rates

The failure of the system or a component is the inability of the system to deliver its intended function satisfactorily. Hazard rate is the instantaneous speed of failures. It is expressed as the ratio of the number of failures in a small interval of time to the product of number of surviving items. Repairs are the process of restoration work of any failed system. The ability of an item, under stated conditions of use, to be retained in, or restored to, a state in which it can perform its required functions.

## 5. Mathematical modelling of the power generation processing unit

**Fig 2:**
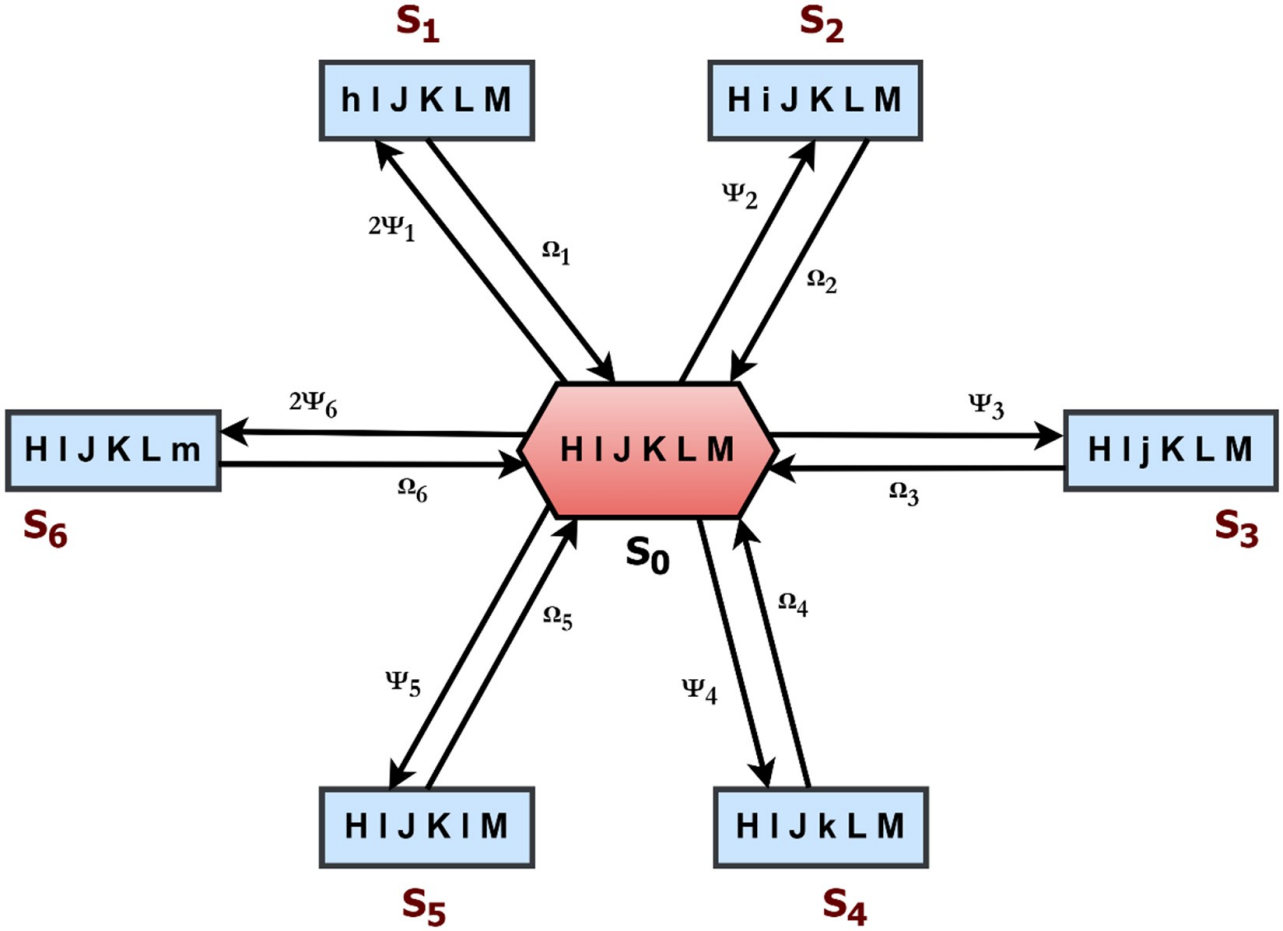
State changeover of the power generation processing system.

The mathematical model of the power generating unit is developed using the Markov birth-death process based on the state changeover diagram (Fig 2) where all repair rates are exponentially distributed. This model is described below:

$$\begin{array}{ll} P_{0}(t+\Delta t)= & \left[(1-2\Psi_{1}-\Psi_{2}-\Psi_{3}-\Psi_{4}-\Psi_{5}-2\Psi_{6})P_{0}(t)+\Omega_{1}P_{1}(t)+\Omega_{2}P_{2}(t\right)\\ & +\Omega_{3}P_{3}(t)+\Omega_{4}P_{4}(t)+\Omega_{5}P_{5}(t)+\Omega_{6}P_{6}(t)]\Delta t \end{array}$$ (1)

$$\begin{array}{ll} P_{0}(t+\Delta t)-P_{0}(t) & =[(-2\Psi_{1}-\Psi_{2}-\Psi_{3}-\Psi_{4}-\Psi_{5}-2\Psi_{6})P_{0}(t)+\Omega_{1}P_{1}(t)\\ & +\Omega_{2}P_{2}(t)+\Omega_{3}P_{3}(t)\;+\Omega_{4}P_{4}(t)+\Omega_{5}P_{5}(t)+\Omega_{6}P_{6}(t)]\Delta t \end{array}$$ (2)

Dividing both sides by Δ*t* and limit Δ*t* → ∞

$$\underset{\Delta t\to 0}{\text{lim}}\frac{P_{0}(t+\Delta t)-P_{0}(t)}{\Delta t}=\underset{\Delta t\to 0}{\text{lim}}[(-2\Psi_{1}-\Psi_{2}-\Psi_{3}-\Psi_{4}-\Psi_{5}-2\Psi_{6})P_{0}(t)+\Omega_{1}P_{1}(t)+\Omega_{2}P_{2}(t)+\Omega_{3}P_{3}(t)+\Omega_{4}P_{4}(t)+\Omega_{5}P_{5}(t)+\Omega_{6}P_{6}(t)]$$

$$\begin{array}{ll} \left[\frac{dP_{0}(t)}{dt}\right]+(2\Psi_{1}+\Psi_{2}+\Psi_{3}+\Psi_{4}+\Psi_{5}+2\Psi_{6})P_{0}(t) & =\Omega_{1}P_{1}(t)+\Omega_{2}P_{2}(t)+\Omega_{3}P_{3}(t)+\Omega_{4}P_{4}(t)\\ & +\Omega_{5}P_{5}(t)+\Omega_{6}P_{6}(t) \end{array}$$

$$\begin{array}{ll} \left[\frac{d}{dt}+2\Psi_{1}+\Psi_{2}+\Psi_{3}+\Psi_{4}+\Psi_{5}+2\Psi_{6}\right]P_{0}(t) & =\Omega_{1}P_{1}(t)+\Omega_{2}P_{2}(t)+\Omega_{3}P_{3}(t)+\Omega_{4}P_{4}(t)\\ & +\Omega_{5}P_{5}(t)+\Omega_{6}P_{6}(t) \end{array}$$ (3)

$$\left[\frac{d}{dt}+\Omega_{1}\right]P_{1}(t)=2\Psi_{1}P_{0}(t)$$ (4)

$$\left[\frac{d}{dt}+\Omega_{2}\right]P_{2}(t)=\Psi_{2}P_{0}(t)$$ (5)

$$\left[\frac{d}{dt}+\Omega_{3}\right]P_{3}(t)=\Psi_{3}P_{0}(t)$$ (6)

$$\left[\frac{d}{dt}+\Omega_{4}\right]P_{4}(t)=\Psi_{4}P_{0}(t)$$ (7)

$$\left[\frac{d}{dt}+\Omega_{5}\right]P_{5}(t)=\Psi_{5}P_{0}(t)$$ (8)

$$\left[\frac{d}{dt}+\Omega_{6}\right]P_{6}(t)=2\Psi_{6}P_{0}(t)$$ (9)

Initial Conditions:

$$P_{0}(0)=1$$

$$P_{i}(0)=0,\;i=1\;\;to\;7,$$ (10)

To calculate long-run availability, we can take $\frac{d}{dt}=0$ as *t* → ∞ and *P*_*i*_(*t*) = *P*_*i*_

From Eqs (1–9), steady-state probabilities are:

$$\begin{array}{lll} P_{1}=\frac{2\Psi_{1}}{\Omega_{1}}P_{0} & P_{2}=\frac{\Psi_{2}}{\Omega_{2}}P_{0} & P_{3}=\frac{\Psi_{3}}{\Omega_{3}}P_{0}\\ P_{4}=\frac{\Psi_{4}}{\Omega_{4}}P_{0} & P_{5}=\frac{\Psi_{5}}{\Omega_{5}}P_{0} & P_{6}=\frac{2\Psi_{6}}{\Omega_{6}}P_{0} \end{array}$$

Using normalized condition Σ *P*_*i*_ = 1

$$P_{0}={\left(1+\frac{2\Psi_{1}}{\Omega_{1}}+\frac{\Psi_{2}}{\Omega_{2}}+\frac{\Psi_{3}}{\Omega_{3}}+\frac{\Psi_{4}}{\Omega_{4}}+\frac{\Psi_{5}}{\Omega_{5}}+\frac{2\Psi_{6}}{\Omega_{6}}\right)}^{-1}$$

long run availability (*A*_*v*_)

$$A_{v}=P_{0}={\left(1+\frac{2\Psi_{1}}{\Omega_{1}}+\frac{\Psi_{2}}{\Omega_{2}}+\frac{\Psi_{3}}{\Omega_{3}}+\frac{\Psi_{4}}{\Omega_{4}}+\frac{\Psi_{5}}{\Omega_{5}}+\frac{2\Psi_{6}}{\Omega_{6}}\right)}^{-1}$$ (11)

## 6. Optimization strategies

In current age scientists consistently trying to develop new designs for existing systems so that maximum output may be extracted with the minimum cost investment. It is also tried those existing systems optimally used for a long duration. The designing and operationality generally involve the tuning of models for physical structures. Optimization can be used to manage the assignments of design, operation, and tuning models systemically. It is a technique used to select the best solution from a set of feasible solutions. In a more elaborated way, it is explained as a technique that finds the set of variables known as decision variables. It provides the optimum objective function value in a search space bounded by constraints and non-negativity conditions. Several statistical techniques exist in the literature, viz. maximum likelihood estimation, method of moments, least-square estimation, Bayes estimation etc., for parameter estimation and optimization, various linear and non-linear programming techniques exist. Linear programming, integer programming, and dynamic programming are a few techniques to find the optimum value of the objective function, but these only provide the local solution. Metaheuristic and evolutionary algorithms are recently developed techniques which show high efficiency in providing the optimal solution to complex real-world problems and are free from the nature of the problem. Recently, these techniques became popular for finding the optimum solution for complex problems. Metaheuristic approaches are classified into three categories: nature, population, and memory. Recently, several metahuristic approachs (Ant Colony Optimization, Neural Networks, Grey Wolf Optimization, Whale Optimization) proposed by researcher to optimize the performance of process industries and showed the applications in their reliability prediction. Though, no work is reported in availability optimization of power generating unit of sewage treatment plants. To fill this research gap in the present analysis an effort is made to optimize the availability of power generating units by using two well-known nature-based algorithms, genetic algorithm (GA) and particle swarm optimization (PSO). These algorithms are not affected by the nonlinearlity and problem size. Here, a population is randomly generated and assigned to each particle. The best solution is attained corresponding to the Pbest and Gbest. The efficiency of the algorithms is statistically tested using the methodology proposed by Derrac et al. [52] (2011). Mathematically, the optimization problem of the proposed model is formulated using availability function (11) as objective function as follows:

Objecive function: Max. *A*_*v*_

$$\text{Subject}\;\text{to}:\left\{\begin{array}{l} \Psi_{i}\geq 0\\ \Omega_{t}\geq 0 \end{array}\right.$$ (12)

Where i = 1,2,3,4,5,6.

### 6.1 Genetic algorithm

**Fig 3:**
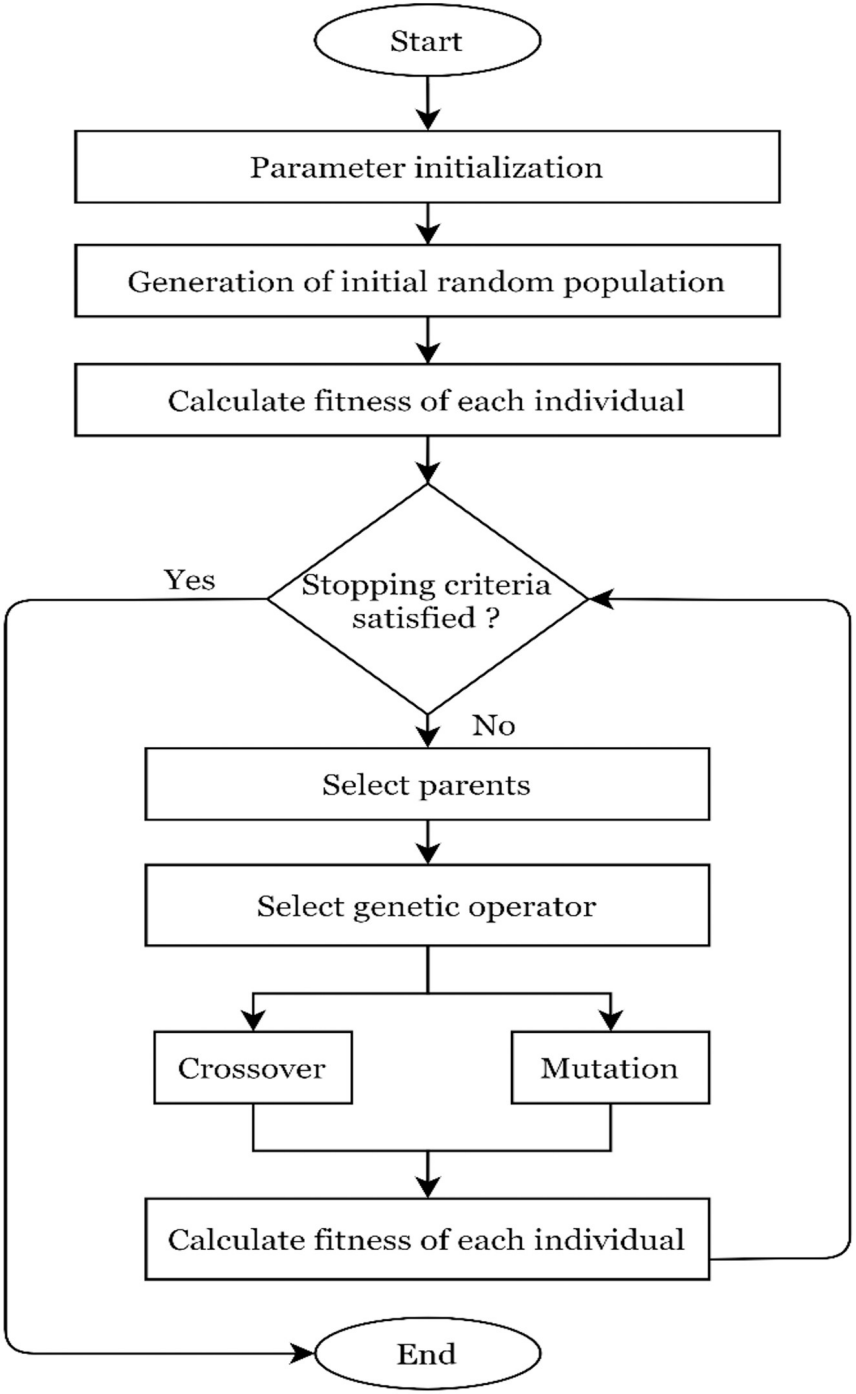
Flowchart of genetic algorithm.

A genetic algorithm (GA) works on the Darwinian theory of survival of the fittest between organisms in danger of extinction by environmental factors and hunters. Goldberg and Holland [53] (1988) developed a genetic algorithm for the first time and used it to find optimal solutions to complex engineering problems. It is based on genetic and natural selection and falls under evolutionary computation. A large population of possible solutions exists in a genetic algorithm, and these solutions undergo crossover and mutation for reproduction. Each possible solution has an assigned fitness value, and better-fitted candidates are chosen for mutation and producing a new generation of solutions. It is observed that the fittest member can easily adopt the changes and have the highest chances of survival. The same characteristics are also followed by their offspring as inherent traits. It resulted in the fittest generations’ production. Moreover, genomic mutations happen randomly among the members of the population, and these also improve the long-term persistence of fit members and their evolutionary progenies. The individual generated through genetic algorithms are known as chromosomes and are treated as a solution to the optimization problem. The chromosome is the combination of genes those stands for decision variables in the optimization problem, and the ability to survive is termed the fitness value of the individual. The surviving individuals of the previous generation and their offspring made the population of each generation. The offspring are generated using genetic operators, namely mutation and crossover. To generate a new generation of solutions, parents are selected, and the probability of selection is proportional to the fitness value of parent. Higher the fitness value resulted in a higher chance of survive. The higher fitness value candidates always get priority over the others. The process goes on until the stopping criteria are satisfied. The flowchart of genetic algorithm is shown in Fig 3. The working pattern of traditional GA is as follows:

Step 1: Generate a random population of possible solutions
Step 2: Calculate the fitness value of each member and select a few as parents based on their fitness value to produce new offspring
Step 3: A new generation of individuals (possible solutions) produced by applying genetic operators’ crossover and mutation.
Step 4: Iteratively, the old individuals are replaced by new individuals, and the process repeatedly continues until the stopping criteria are satisfied.
Step 5: Go to step 2 if the stopping criteria are not satisfied

#### 6.1.1. Crossover

It is one of the essential operators among genetic operators. The new offspring is generated by crossover through the exchange of genes between parents. It is applied to two solutions/parents. Under crossover operation, few decision variables of both solutions are exchanged, i.e., in newly developed solutions, few decision variables come from the first solution, and the rest comes from the second. There are three types of crossover patterns observed: one-point crossover, two-point crossover and uniform crossover. A random crossover point is used in a one-point crossover, and a child is reproduced having some genes of the parent located on one side of the crossover point and ret comes from the parent on the other side. In the two-point crossover, two points are considered, and solutions are generated by the crossover of the parents located outside the crossover points. All the parent solution within crossover points is protected. In uniform, crossover points are randomly generated. In the present analysis, uniform crossover generates the new population.

#### 6.1.2. Mutation

It provides new genetic material for the population. It generally changes some genes of the offspring. It replaces some decision variable of the new solution with any random number while other decision variables remain unchanged. Mutations are of two kinds, viz. uniform and non-uniform. Uniform mutation replaces the gene with another gene from the feasible solution space. Non-uniform mutation introduces a localized search space for an optimal solution where sets of decision variables/genes are based on the boundaries. These boundaries shrink with the increasing number of iterations of GA.

### 6.2 Particle Swarm Optimization (PSO)

**Fig 4:**
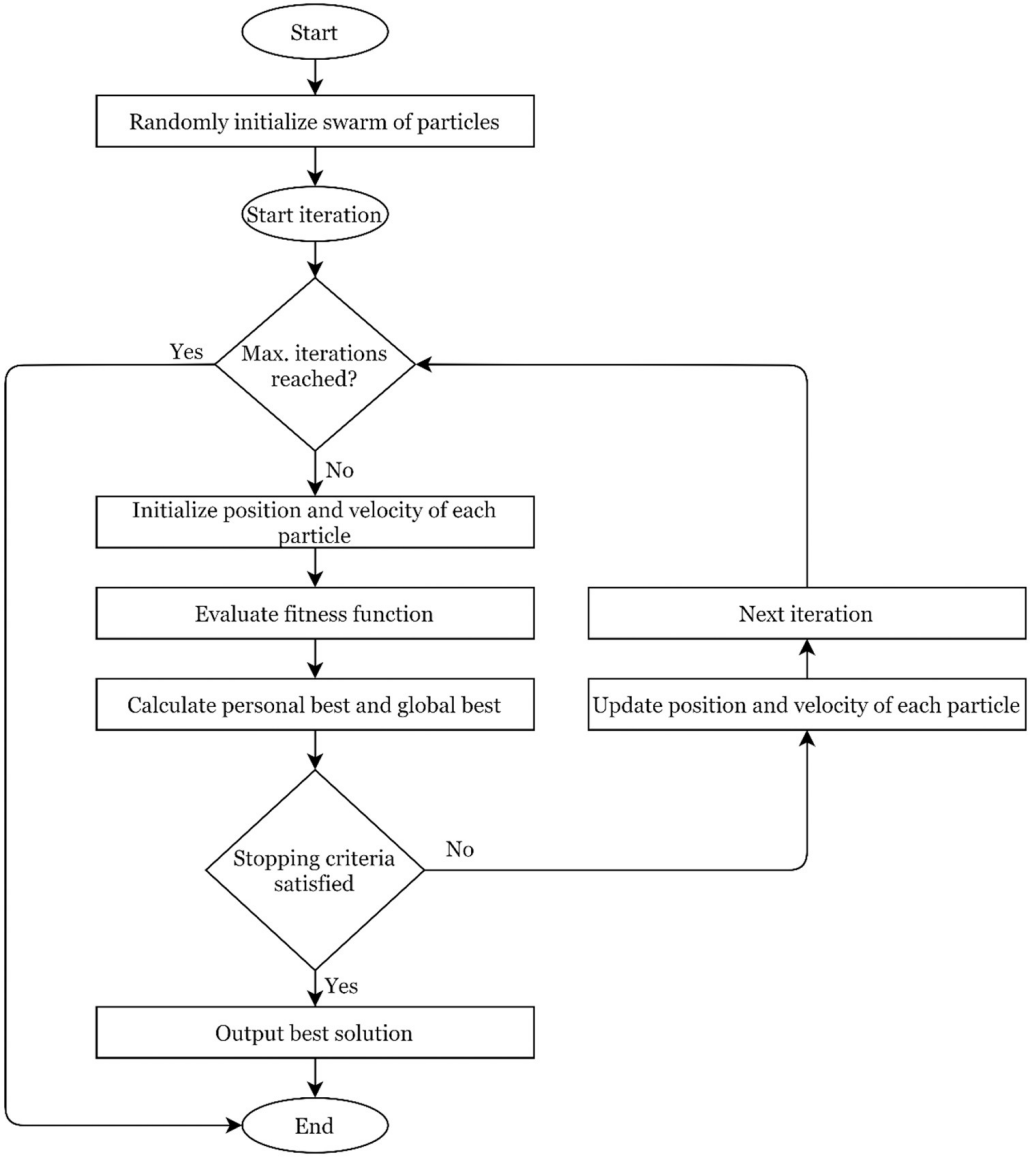
Flowchart of particle swarm optimization algorithm.

Computational intelligence techniques based on swarm behaviour gained popularity during the last few decades. The methods based on animals’ social and biological behaviour collectively and individually when interacting with each other or with the environment are termed swarm-based, and it is termed swarm intelligence. In swarm intelligence, a group of individuals/solutions handles real-world systems by ordinating themselves by self-discipline and decentralization. Particle swarm optimization (PSO) is a well-known example of swarm-based computational intelligence technique. It is worked on the social behaviour of birds and replicates the behaviour of the herds. In this technique, initially, it is assumed that a herd of birds is looking for food, and no information is available for the food. As an effective strategy, the herd follow the bird, which knows the nearest food source. The PSO works on the same approach and utilizes an initial numerical solution from the search space to optimize the problem’s solution. Each solution is termed a bird in an optimization problem and a particle. The set of particles is known as swarm. The particles have a fitness value derived using an objective function and a velocity with which particles move in the problem’s search space. A chief guides all the particles in the search space. The particles change their position based on their personal best position as well as group best position. The flowchart of particle swarm optimization is shown in Fig 4.

The implementation criteria of PSO are described as follows:

Step 1: Input the initial numerical velocity and acceleration values of all the particles from the solution search space.
Step 2: Calculate the fitness value using the objective function of the problem for all the particles. These derived values are the best personal position and fitness values achieved. The best position among all the particles is termed as global best position.
Step 3: The new solutions are generated by updating the position and velocity based on personal and global best.
Step 4: The next iteration is started; fitness values are recalculated, and personal and global best positions are updated.
Step 5: If convergence criteria are met, stop; otherwise, go back to step 3.

## 7. Implementation of optimization strategies

In complex industrial systems like sewage treatment plants, many components are involved, and their global solution is impossible to achieve. So, here in this situation use of metaheuristic approaches is recommended. The performance of the sewage treatment plant is highly influenced by its subsystems’ failure and repair rate. The failure and repair rate parameter constraints for the power generation processing unit are as follows: (Ψ_1_, Ω_1_), (Ψ_2_, Ω_2_), (Ψ_3_, Ω_3_), (Ψ_4_, Ω_4_), (Ψ_5_, Ω_5_), (Ψ_6_, Ω_6_).

The range of parameter constraints are: Ψ_1_ ∈ [0.001, 0.02], Ψ_2_ ∈ [0.0003, 0.006], Ψ_3_ ∈ [0.0002, 0.04], Ψ_4_ ∈ [0.0005, 0.07], Ψ_5_ ∈ [0.0001, 0.009], Ψ_6_ ∈ [0.0023, 0.075], Ω_1_ ∈ [0.89, 2.35], Ω_2_ ∈ [0.02, 1.34], Ω_3_ ∈ [0.0032, 3.45], Ω_4_ ∈ [0.56, 2.33], Ω_5_ ∈ [0.063, 1.89], Ω_6_ ∈ [2.77, 4.98]. The range of failure and repair rates is taken arbitrarly after investigating various studies.

In the present study, the long-run availability of the power generation processing unit is optimized by applying genetic algorithm and Particle swarm optimization on the optimization model appended in Eq (12).

In implementing GA, five steps are mainly involved, namely encoding, fitness function, selection, mutation, and crossover. Here, value encoding is used to encode the chromosome values; a random selection technique is used to select the parent population to operate crossover. Here, uniform crossover and mutation are used to generate new offspring. As an illustration, all the five steps are explained below:

(i) Encoding

Encoding is an operator in GA used for mapping chromosomes, a set of different values. It is highly dependent on the objective function of the study. A sample chromosome value is given as below:

Power generation processing unit

Ψ_1_ = 0.0006, Ψ_2_ = 0.0014, Ψ_3_ = 0.0033, Ψ_4_ = 0.0089, Ψ_5_ = 0.0004, Ψ_6_ = 0.0015, Ω_1_ = 1.78, Ω_2_ = 0.75, Ω_3_ = 2.45, Ω_4_ = 1.89, Ω_5_ = 0.075, Ω_6_ = 3.25

(ii) Fitness Function

Fitness function is described as the obtained solution quality near the prescribed set of values. A sample chromosome with a fitness value is given as below:

Power generation processing unit

Ψ_1_ = 0.006, Ψ_2_ = 0.0014, Ψ_3_ = 0.0033, Ψ_4_ = 0.0089, Ψ_5_ = 0.0004, Ψ_6_ = 0.015, Ω_1_ = 1.78, Ω_2_ = 0.75, Ω_3_ = 2.45, Ω_4_ = 1.89, Ω_5_ = 0.075, Ω_6_ = 3.25

The fitness value of availability for power generating units is 0.9977.

(iii) Selection: Here, the parent population are selected randomly to generate an offspring population.

(iv) Crossover: Cross-over operator is a technique in which the child population is generated from the paired parent population. Here the uniform method of crossover is applied.

Power generation processing unit

Ψ_1_ = 0.0034, Ψ_2_ = 0.0016, Ψ_3_ = 0.0009, Ψ_4_ = 0.0027, Ψ_5_ = 0.0004, Ψ_6_ = 0.0154, Ω_1_ = 5.0452, Ω_2_ = 0.5375, Ω_3_ = 3.6653, Ω_4_ = 0.4712, Ω_5_ = 0.1761, Ω_6_ = 3.9891

(v) Mutation: The mutation operator is useful to maintain the diversity from one generated population to the next.

Power generation processing unit

Ψ_1_ = 0.0130, Ψ_2_ = 0.0004, Ψ_3_ = 0.0018, Ψ_4_ = 0.0005, Ψ_5_ = 0.0000, Ψ_6_ = 0.0047, Ω_1_ = 2.2237, Ω_2_ = 0.9349, Ω_3_ = 0.7339, Ω_4_ = 0.8068, Ω_5_ = 0.2604, Ω_6_ = 5.6486

The results of genetic algorithm appended in Tables 1–4. From Table 1, it is revealed that at mutation probability 0.55, crossover probability 0.62, number of evaluations 200, the maximum availability of power generating unit is 0.9980, corresponding to population size 10. The estimated parameters values given against the population zsize 10.

**Table 1:** Effect of population size on availability of power generation processing unit by using Genetic Algorithm (Evolution = 200, Mutation = 0.55, Crossover = 0.62).

Pop Size	10	20	30	40	50	60	70	80
**Availability**	**0.998**	0.9976	0.997	0.9962	0.9962	0.9958	0.997	0.9974
*Ψ*_1_	**0.0127**	0.0105	0.0096	0.0055	0.0001	0.0023	0.005	0.0095
*Ψ*_2_	**0.0005**	0.0014	0.0006	0.0001	0.0008	0.0003	0.0003	0
*Ψ*_3_	**0.006**	0.0014	0.0073	0.0001	0.0016	0.0023	0.006	0.0022
*Ψ*_4_	**0.0189**	0.0041	0.0011	0.0064	0.0348	0.0016	0.0097	0.0014
*Ψ*_5_	**0.0002**	0.0001	0.0002	0.00000	0.00000	0.0001	0.0001	0.0009
*Ψ*_6_	**0.029**	0.0059	0.0037	0.0393	0.0072	0.0018	0.0049	0.0087
Ω_1_	**1.0735**	5.6052	0.9193	2.9175	1.5169	2.9504	5.6678	2.9458
Ω_2_	**1.5022**	0.9036	1.3094	4.8099	1.8135	1.9539	1.4146	3.319
Ω_3_	**2.3254**	1.7609	6.1739	0.3296	3.0717	1.5157	2.3848	1.1834
Ω_4_	**3.1476**	2.0172	0.355	0.2843	2.6589	0.6751	2.0006	2.4417
Ω_5_	**0.1985**	0.1937	0.1406	0.2916	0.1227	0.3169	0.0814	0.07
Ω_6_	**3.6401**	3.5426	9.0641	15.9506	6.5042	2.5655	3.4049	6.9542

**Table 2:** Effect of evolution on availability of power generation processing unit by using Genetic Algorithm (Population size = 60, Mutation = 0.55, Crossover = 0.62).

Evolution	25	50	75	100	125	150	175	200
**Availability**	**0.9972**	0.9964	0.9964	0.996	0.9963	0.9957	0.9942	0.9968
*Ψ*_1_	**0.0009**	0.0009	0.0104	0.0092	0.0019	0.0016	0.0008	0.0024
*Ψ*_2_	**0.001**	0.0001	0.0003	0.001	0.0017	0.0003	0.0012	0.0009
*Ψ*_3_	**0.0006**	0.0036	0.0009	0.0036	0.0036	0.0006	0.0014	0.0105
*Ψ*_4_	**0.0139**	0.0054	0.0006	0.0016	0.0027	0.0052	0.0022	0.0083
*Ψ*_5_	**0.0002**	0.0001	0.0009	0	0	0.0002	0.0006	0.0009
*Ψ*_6_	**0.0069**	0.0104	0.0255	0.0053	0.0054	0.0082	0.0088	0.041
Ω_1_	**1.1273**	6.2075	4.1148	2.8301	4.2875	3.7348	2.6192	3.6029
Ω_2_	**0.3649**	0.741	0.5354	0.7667	0.5651	1.6236	3.7308	1.6457
Ω_3_	**2.4867**	2.1293	4.0273	0.7512	1.1112	0.667	0.6511	9.3568
Ω_4_	**3.6797**	3.5884	0.2078	0.779	1.2169	2.6521	7.228	3.8126
Ω_5_	**0.0536**	0.1293	0.1734	0.0853	0.1467	0.1436	0.07	0.053
Ω_6_	**3.3547**	3.5199	10.1394	7.2158	4.1896	3.4379	4.4726	14.1331

**Table 3:** Effect of crossover on availability of power generation processing unit by using Genetic Algorithm (Population size = 60, Evolution = 200, Mutation = 0.55).

Crossover	0.1	0.2	0.3	0.4	0.5	0.6	0.7	0.8
**Availability**	0.9965	0.9975	0.9965	0.9955	0.9975	0.9943	0.9836	**0.9982**
*Ψ*_1_	0.0055	0.0038	0.0044	0.0148	0.0034	0.0029	0.0001	**0.003**
*Ψ*_2_	0.0016	0.0009	0.0003	0.0018	0.0016	0.0007	0.0006	**0.0033**
*Ψ*_3_	0.0029	0.0069	0.0004	0.0036	0.0009	0.0036	0.0009	**0.002**
*Ψ*_4_	0.0051	0.0026	0.0095	0.0002	0.0027	0.0003	0.0041	**0.0193**
*Ψ*_5_	0.0004	0	0.0003	0.0002	0.0004	0.0004	0.0001	**0.0002**
*Ψ*_6_	0.0101	0.0017	0.0107	0.0084	0.0154	0.0075	0.0354	**0.0019**
Ω_1_	2.4431	3.4783	4.2274	5.9378	5.0452	6.313	8.8967	**2.1281**
Ω_2_	1.2373	2.0046	3.0758	0.5594	0.5375	0.4693	1.272	**2.4387**
Ω_3_	0.9766	2.1187	2.4587	0.2759	3.6653	0.4525	5.3047	**1.2429**
Ω_4_	2.9661	3.1495	1.6219	4.5832	0.4712	6.0316	13.2156	**2.1461**
Ω_5_	0.1012	0.0289	0.1478	0.1808	0.1761	0.0666	0.2988	**0.1605**
Ω_6_	5.1355	13.8018	5.5587	7.5367	3.9891	5.4234	2.5219	**3.7112**

**Table 4:** Effect of mutation on availability of power generation processing unit by using Genetic Algorithm (Population size = 60, Evolution = 200, Crossover = 0.62).

Mutation	0.1	0.2	0.3	0.4	0.5	0.6	0.7	0.8
**Availability**	0.997	0.9963	**0.9977**	0.9949	0.9975	0.9961	0.9967	0.9958
*Ψ*_1_	0.0002	0.0014	**0.003**	0.0126	0.013	0.0022	0.0071	0.0058
*Ψ*_2_	0.0002	0.0014	**0**	0.0004	0.0004	0.0006	0.0012	0.0011
*Ψ*_3_	0.0016	0.0065	**0.0015**	0.0017	0.0018	0.0017	0.0001	0.0055
*Ψ*_4_	0.005	0.0108	**0.0098**	0.0016	0.0005	0.0062	0.0194	0.0023
*Ψ*_5_	0.0003	0.0002	**0.0007**	0.00000	0.000000	0.0001	0.0002	0.0006
*Ψ*_6_	0.0027	0.021	**0.0008**	0.0029	0.0047	0.0274	0.0066	0.0044
Ω_1_	3.3911	4.6373	**1.631**	1.4176	2.2237	6.1526	5.8459	2.102
Ω_2_	1.5723	0.7252	**1.0961**	1.8721	0.9349	0.8043	1.3065	1.3479
Ω_3_	6.3796	1.4625	**2.7384**	0.9221	0.7339	0.4638	1.9506	2.4403
Ω_4_	1.4828	2.0897	**1.9353**	5.4195	0.8068	1.7697	8.1885	2.5528
Ω_5_	0.1689	0.1762	**0.2052**	0.1125	0.2604	0.1338	0.1981	0.0449
Ω_6_	4.9002	6.184	**2.8777**	5.7425	5.6486	17.4137	6.6902	7.2804

From Table 2, it is revealed that at mutation probability 0.55, crossover probability 0.62, and population size 60, the maximum availability of power generating unit is 0.9972 after 25 evolutions. Table 3 revealed that at mutation probability 0.55, population size 60, and the number of evaluations 200, the maximum availability of power generating unit is 0.9982, corresponding to crossover probability 0.8.

Table 4 revealed that at crossover probability 0.62, population size 60, and the number of evaluations 200, the maximum availability of power generating unit is 0.9977, corresponding to mutation probability 0.3.

**Fig 5:**
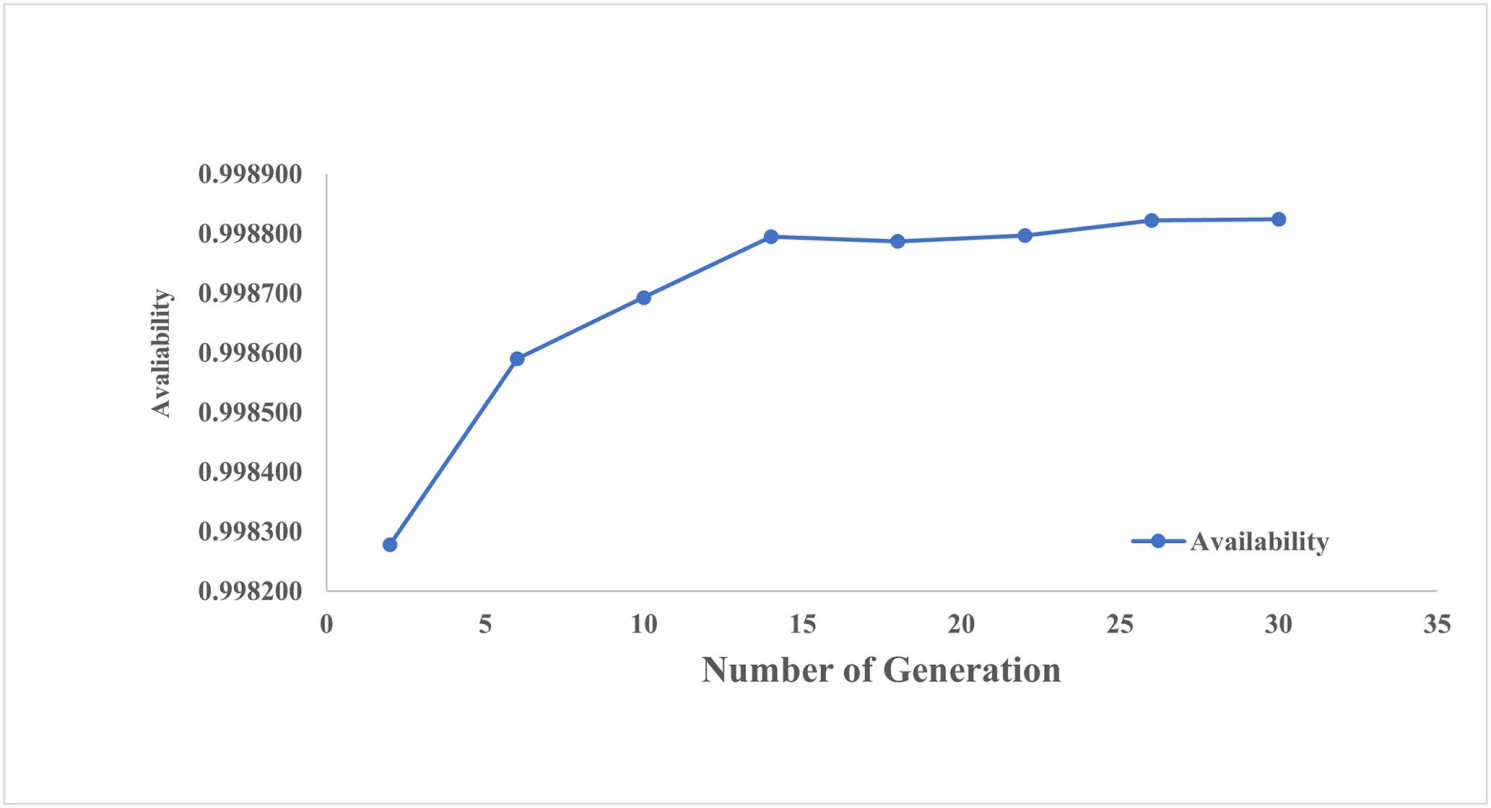
No. of generation vs. availability.

It is observed that optimum availability using a genetic algorithm can be achieved at a crossover probability of 0.8, a population size of 60, mutation probability of 0.55, and a number of evaluations of 200. The implementation of particle swarm optimization generated numerical results in various situations. The numerical results with respect to number of generations, number of iterations and damping ration are appended in Tables 5–7. From Table 5, it is revealed that after 30 generations, availability got optimized corresponding to population size 15, inertia weight 1, damping ratio 0.95, p-best 1.7 and g-best 2.3. Fig 5 showed the pattern of availability along with the number of generations.

**Table 5:** Steady-state availability with respect to the number of generations by using Particle Swarm Optimization having a population size of 15, inertia weight 1, damping ratio 0.95, p-best 1.7 and g-best 2.3.

Number of Generation	2	6	10	14	18	22	26	30
**Availability**	0.998278	0.99859	0.998693	0.998795	0.998787	0.998797	0.998822	**0.998824**
*Ψ*_1_	0.0031	0.0011	0.0135	0.0019	0.0026	0.013	0.0044	**0.002**
*Ψ*_2_	0.0031	0.0013	0.0039	0.0039	0.006	0.0029	0.0033	**0.0028**
*Ψ*_3_	0.019	0.0308	0.023	0.0137	0.0177	0.0016	0.007	**0.0173**
*Ψ*_4_	0.0378	0.0127	0.0144	0.0009	0.0182	0.0361	0.0011	**0.0011**
*Ψ*_5_	0.0084	0.0046	0.0037	0.0083	0.0066	0.0001	0.0084	**0.0072**
*Ψ*_6_	0.0068	0.0237	0.023	0.0162	0.0304	0.0035	0.0134	**0.0206**
Ω_1_	1.0131	2.1649	2.1078	1.76	1.7486	1.7566	2.3138	**0.9916**
Ω_2_	1.0786	1.107	1.0197	1.0805	1.0464	0.9585	0.6786	**1.2462**
Ω_3_	1.7587	3.4459	2.9878	0.7857	2.4551	0.8066	3.2497	**2.6792**
Ω_4_	1.2499	1.2229	1.298	2.0864	2.2364	1.9217	1.9417	**1.368**
Ω_5_	0.2799	0.9913	1.4196	1.6971	1.4516	0.506	1.2887	**1.2856**
Ω_6_	4.2181	4.4242	2.9246	3.3431	3.1561	4.2463	3.4643	**3.4456**

**Table 6:** Steady-state availability with respect to the number of iterations by using Particle Swarm Optimization having population size 15, inertia weight 1, damping ratio 0.95, p-best 1.7 and g-best 2.3.

Number of Iteration	4	8	12	16	20	24	28	32
**Availability**	0.998133	0.998491	0.998524	0.998522	0.998685	0.998781	0.998807	**0.998815**
*Ψ*_1_	0.0085	0.0119	0.0012	0.0168	0.0149	0.0071	0.0096	**0.0124**
*Ψ*_2_	0.0048	0.0056	0.0022	0.0027	0.0026	0.0008	0.0055	**0.0016**
*Ψ*_3_	0.0112	0.0355	0.0082	0.0352	0.0126	0.0008	0.023	**0.0218**
*Ψ*_4_	0.0233	0.0102	0.0147	0.0162	0.0015	0.0023	0.0153	**0.0022**
*Ψ*_5_	0.0018	0.0041	0.0014	0.0005	0.0026	0.0027	0.0033	**0.002**
*Ψ*_6_	0.0035	0.0222	0.0221	0.0049	0.0043	0.0141	0.0137	**0.0046**
Ω_1_	2.2763	2.1531	2.1982	1.7996	2.2958	0.9954	1.6352	**1.4503**
Ω_2_	0.6964	0.6477	1.0184	0.8128	1.3144	1.3098	1.2939	**0.8476**
Ω_3_	2.5377	2.3623	2.3549	2.6077	3.1127	0.2992	2.4255	**2.9288**
Ω_4_	1.4819	1.7627	1.5912	1.6122	1.4872	1.9699	1.3984	**2.3168**
Ω_5_	0.6329	1.4521	1.0367	1.0112	0.8428	0.9551	1.8472	**0.5395**
Ω_6_	4.9633	4.0621	3.6132	4.8277	3.7118	3.2909	4.6011	**4.179**

**Table 7:** Steady-state availability with respect to weight damping ratio by using Particle Swarm Optimization having maximum iterations 25, population size 15, inertia weight 1, damping ratio 0.95, p-best 1.7 and g-best 2.3.

Weight Damping Ratio	0.1	0.2	0.3	0.4	0.5	0.6	0.7	0.8
**Availability**	0.998671	**0.998816**	0.998721	0.998725	0.998782	0.9882	0.998811	0.998814
*Ψ*_1_	0.012	**0.0057**	0.0081	0.0046	0.0011	0.0091	0.013	0.0015
*Ψ*_2_	0.0019	**0.0053**	0.0023	0.0008	0.0043	0.005	0.0031	0.0026
*Ψ*_3_	0.0342	**0.0038**	0.005	0.0103	0.0065	0.0114	0.0247	0.0054
*Ψ*_4_	0.0199	**0.001**	0.0087	0.02	0.0047	0.0365	0.0168	0.015
*Ψ*_5_	0.0042	**0.0031**	0.0074	0.0062	0.0003	0.0018	0.0089	0.008
*Ψ*_6_	0.0514	**0.042**	0.043	0.007	0.0393	0.0055	0.0057	0.031
Ω_1_	2.3404	**2.2519**	2.2981	1.8646	2.0373	2.0369	1.4393	2.1507
Ω_2_	0.4296	**0.6124**	1.1407	0.4571	1.3332	1.3041	0.967	1.2007
Ω_3_	3.1084	**2.5448**	0.534	1.2526	3.0957	2.2273	1.4881	2.8502
Ω_4_	1.2317	**1.2969**	1.4866	1.9981	0.8509	2.2095	1.6743	1.9802
Ω_5_	1.5044	**1.2759**	0.1194	0.3214	0.3647	0.8488	1.2081	1.773
Ω_6_	4.5983	**3.727**	3.3639	2.9108	4.9057	4.9032	4.825	3.9207

**Fig 6:**
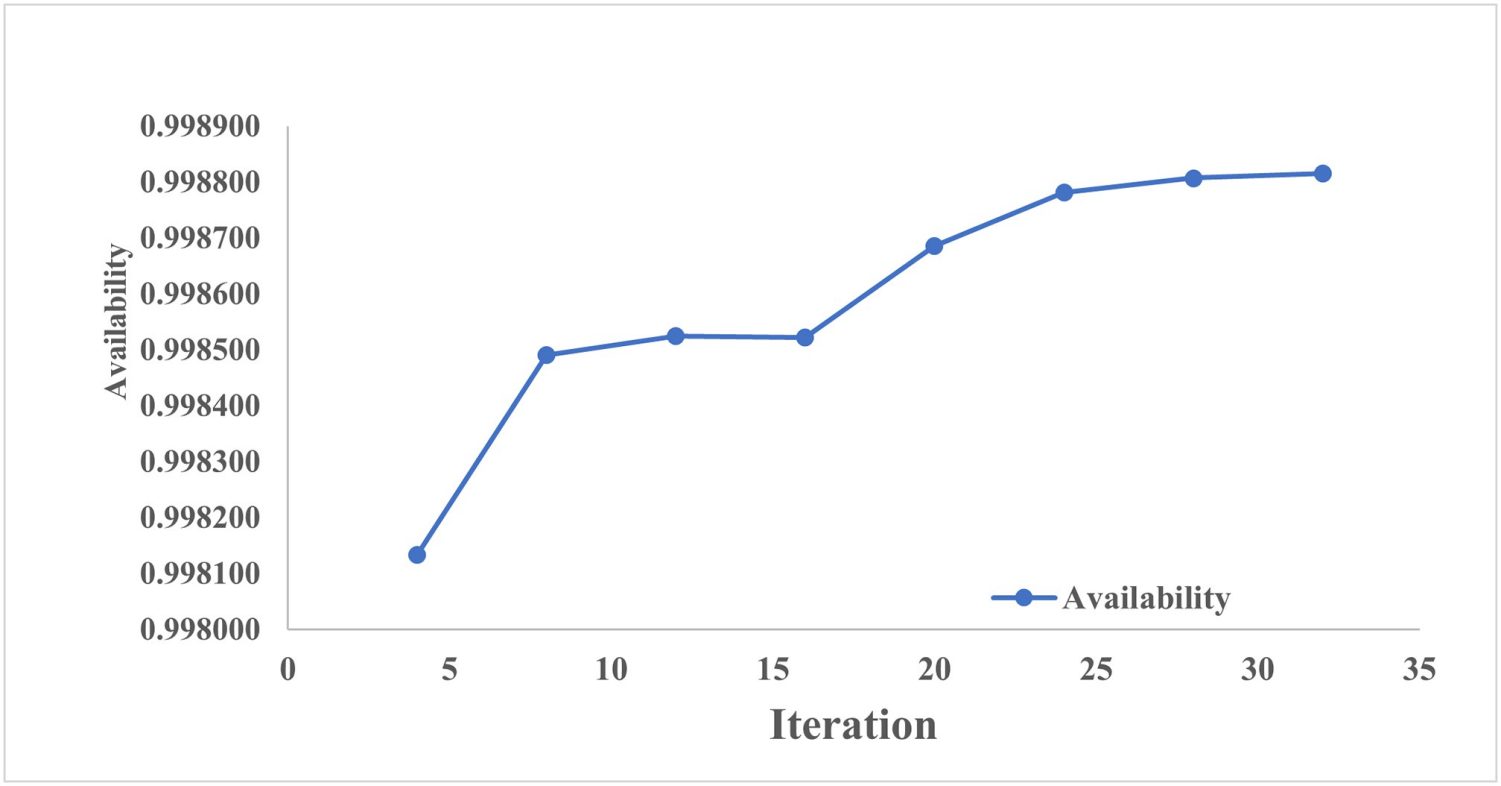
No. of iterations vs. availability.

From Table 6, it is revealed that after 32 iterations, availability got optimized corresponding to population size 15, inertia weight 1, damping ratio 0.95, p-best 1.7 and g-best 2.3. Fig 6 showed the pattern of availability along with the number of iterations.

**Fig 7:**
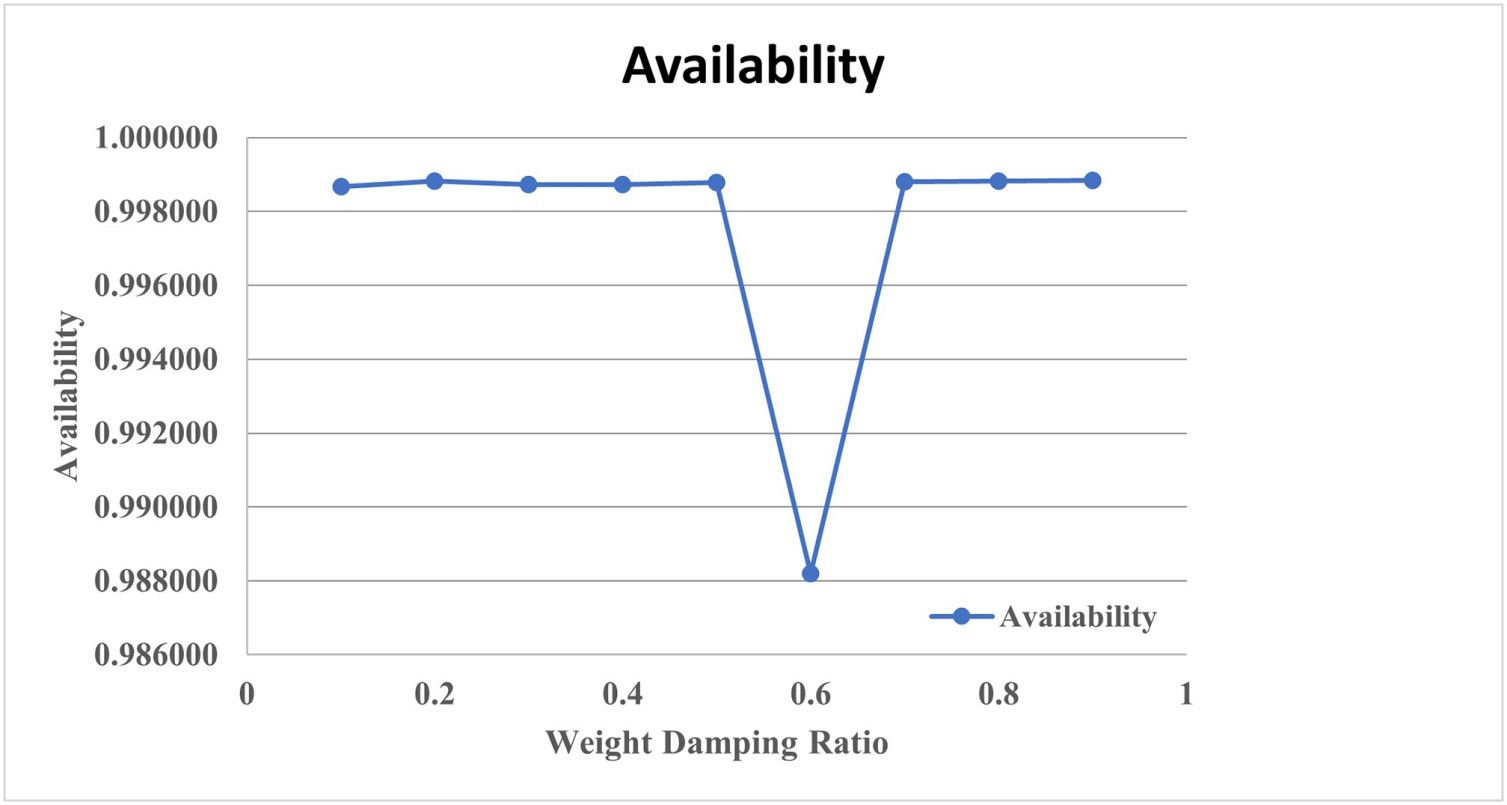
Damping ratio vs. availability.

**Fig 8:**
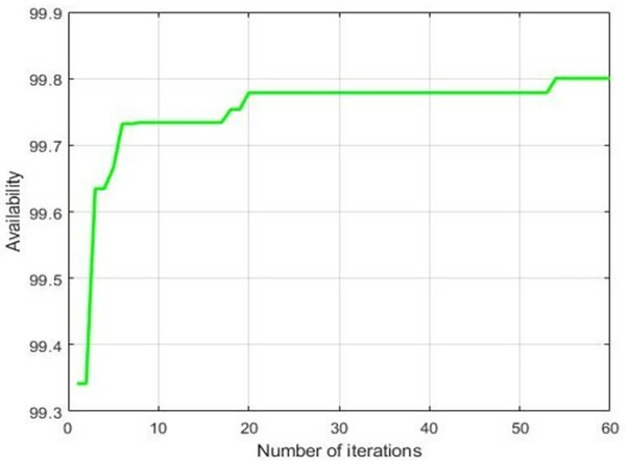
Convergence of availability using GA.

**Fig 9:**
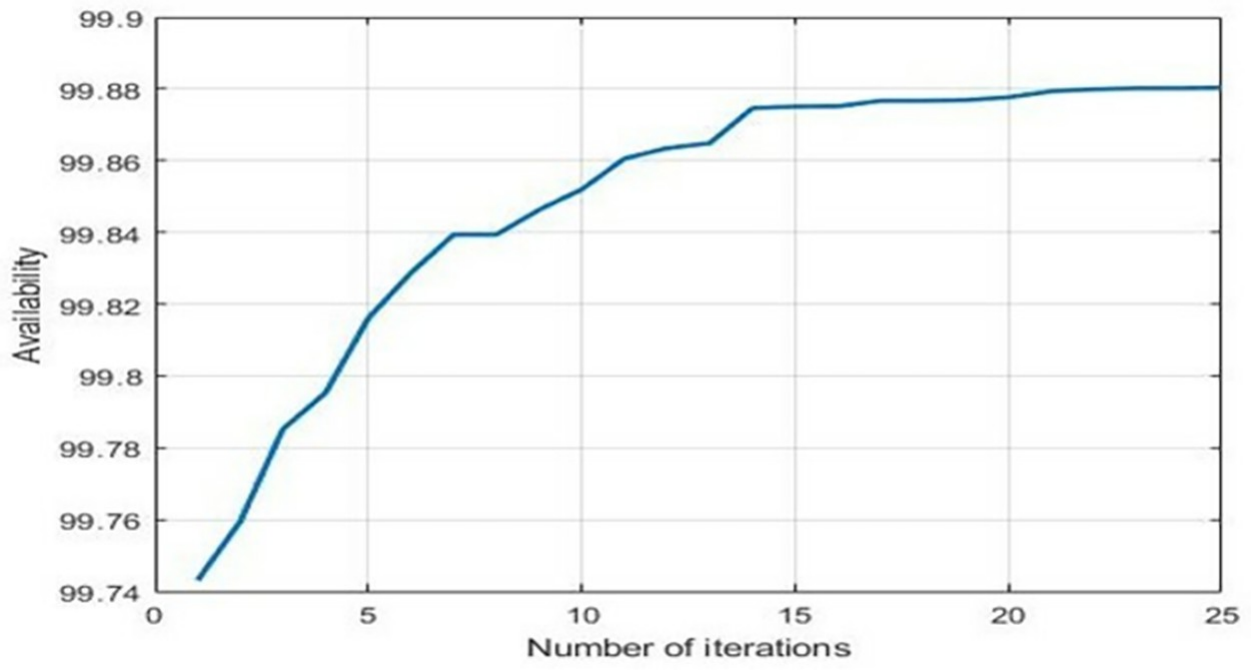
Convergence of availability using PSO.

From Table 7, it is revealed that at weight damping ratio 0.95, availability got optimized corresponding to population size 15, inertia weight 1, maximum iteration 25, p-best 1.7 and g-best 2.3. Fig 7 showed the pattern of availability along with the damping ratio. Figs 8 and 9 revealed the convergence pattern of the availability function using GA and PSO.

After applying Mann-Whitney U-test on the availability of GA and PSO, the following statistics were obtained: *z-*value = 3.363 and U statistics = 0.0001. At 5% level of significance, our *z* critical value is 1.96. Herewith, *z* calculated is greater than z critical value, so we reject the null hypothesis that the performance of both algorithms is equal. It is concluded that Particle Swarm Optimization algorithm outperforms on Genetic algorithm in the prediction of optimal availability of power generating unit of sewage treatment plant.

## 8. Conclusion

In present study, an effort is made to predict the optimal availability of power generating unit of sewage tretatment plant using genetic algorithm and particle swarm optimization. For this purpose, a stochastic model of power generating unit is proposed. The numerical result for the proposed model is derived and optimized. For the power generation processing unit, simulation is done for the population size, which varies from 10 to 80. The maximum availability value is 0.9980, corresponding to the population size of 10 in the considered range of population size. The evaluation varies from 25 to 225 maximum availability value is 0.9972, corresponding to the evaluation value equal to 25. The maximum availability achieved corresponding to crossover and mutation is 0.9982 at 0.8 and 0.9977, corresponding to the mutation value 0.3. Finally, after observing all the derived results, it is revealed that genetic algorithm predicts the maximum availability of power generation processing unit 0.9982 at population size 60, evolution 200, mutation probability 0.55 and crossover probability 0.8. The best-fitted parameter values of failure and repair rates are also derived. After observing particle swarm optimization results, it is identified that the predicted optimum availability value is 0.998833, corresponding to the maximum number of iterations 25, population size 15, inertia weight 1, damping ratio 0.95, p-best 1.7 and g-best 2.3. The statistical investigation of GA and PSO results justified that PSO outperforms than GA. The proposed model and result will be helpful to system developers in designing the subsystems of the sewage treatment plant and establishing new plants. The proposed methodology and algorithms can be utilized in other production and process industries like Paper and Pulp, Shoe Manufacturing, Sugar Industry, Sewage Treatment Plant, etc., to optimize the performance of the systems. Some new optimization algorithms like Differential evolution [54] Ant colony optimization [55], Grey wolf optimizer [56]; Elephant herding optimization [57]; Moth search algorithm [58], Earthworm optimization algorithm [59], Monarch butterfly optimization [60], Harris hawks optimization [61], and Slime mould algorithm [62] as future work to investigate more accurately the performance of the availability of power generating unit. The present model is proposed under the assumptions that failure and repair are constantly distributed, no simultaneous failures and availability of sufficient repair faicilities. These can be observed as the limitations of the present work and system performs better under these conditions. Though the present study is conducted on a medium size sewage treatment plant it can not be established in the entities like small industries, residential societies etc. So, there is a need to explore the possibility of establishment of large size and more complex sewage treatment plants and their performance evaluation. The analysis of large size and more complex sewage treatment plants can be done in future work.

## Abbreviations

*P*_0_(*t*): Probability that system is working with full capacity
Ψ_*i*_(1≤*i*≤6): Respectively failure rates in subsystems *H*, *I*, *J*, *K*, *L*, *M*
Ω_*i*_(1≤*i*≤6): Respectively repair rate in subsystems *H*, *I*, *J*, *K*, *L*, *M*
*h*, *i*, *j*, *k*, *l*, *m*: Subsystem has failed
*H*, *I*, *J*, *K*, *L*, *M*: All subsystems are working with full capacity
*P*_*i*_(*t*), (*i* = 1,‥‥…, 6): Probability of subsystem on i^th^ state at time t
*S*_*i*_, (*i* = 0,1,2,‥‥…, 6): State of the subsystem
System is working with full capacity
System is in failure state
